# Safety and efficacy of bilateral staged focused ultrasound thalamotomy in refractory essential tremor

**DOI:** 10.1093/braincomms/fcaf168

**Published:** 2025-05-02

**Authors:** Marina Campins-Romeu, Rebeca Conde-Sardón, Isabel Sastre-Bataller, Raquel Baviera-Muñoz, Mireya Losada-López, Carlos Morata-Martínez, María José Ibáñez-Juliá, José Luís León-Guijarro, Julia Pérez-García, Luis Raga-Rodríguez, Andrés M Lozano, Antonio Gutiérrez-Martín, Irene Martínez-Torres

**Affiliations:** Movement Disorders Unit, Department of Neurology, University and Polytechnic La Fe Hospital, Valencia 46026, Spain; Unit of Functional Neurosurgery, Department of Neurosurgery, University and Polytechnic La Fe Hospital, Valencia 46026, Spain; Neuroscience Department Ascires Biomedical Group, Valencia 46004, Spain; Movement Disorders Unit, Department of Neurology, University and Polytechnic La Fe Hospital, Valencia 46026, Spain; Movement Disorders Unit, Department of Neurology, University and Polytechnic La Fe Hospital, Valencia 46026, Spain; Cellular, Molecular and Genomics Biomedicine Group, Hospital La Fe Health Research Institute, Valencia 46026, Spain; Neuroscience Department Ascires Biomedical Group, Valencia 46004, Spain; Movement Disorders Unit, Department of Neurology, University and Polytechnic La Fe Hospital, Valencia 46026, Spain; Neuroscience Department Ascires Biomedical Group, Valencia 46004, Spain; Neuroscience Department Ascires Biomedical Group, Valencia 46004, Spain; Movement Disorders Unit, Department of Neurology, University and Polytechnic La Fe Hospital, Valencia 46026, Spain; Research Group in Alzheimer Disease, Hospital La Fe Health Research Institute, Valencia 46026, Spain; Neuroscience Department Ascires Biomedical Group, Valencia 46004, Spain; Division of Neurosurgery, Department of Surgery, University of Toronto, Toronto, Ontario, Canada M5T 2S8; Unit of Functional Neurosurgery, Department of Neurosurgery, University and Polytechnic La Fe Hospital, Valencia 46026, Spain; Neuroscience Department Ascires Biomedical Group, Valencia 46004, Spain; Movement Disorders Unit, Department of Neurology, University and Polytechnic La Fe Hospital, Valencia 46026, Spain; Neuroscience Department Ascires Biomedical Group, Valencia 46004, Spain

**Keywords:** focused ultrasound, essential tremor, thalamotomy, bilateral, staged

## Abstract

Essential Tremor is a common movement disorder characterized by postural and kinetic tremor, primarily affecting the upper limbs, head and voice. For patients who fail medical therapy, neurosurgical interventions such as thalamotomy have been explored. This study evaluates the efficacy and safety of bilateral staged Magnetic Resonance Imaging-guided High-Intensity Focused Ultrasound thalamotomy for the treatment of medication-refractory Essential Tremor. From January 2022 to January 2024, 20 patients who had previously undergone successful unilateral focused ultrasound thalamotomy were enrolled. The primary outcome was the change in tremor severity, using the Clinical Rating Scale for Tremor at 6 months post-second side thalamotomy. Secondary outcomes included functional disability, quality of life and adverse events, particularly balance and gait impairments. Results demonstrated significant tremor reduction, with a 59.98% decrease in Clinical Rating Scale for Tremor A + B score from baseline to 6 months after the second thalamotomy. Quality of life also improved markedly, with an 84.91% reduction in disability and significant enhancement in physical and psychosocial aspects of quality of life. Adverse events were predominantly mild; with subjective gait instability and paresthaesia being the most common. Notably, no cases of severe ataxia or cognitive impairment were observed. Bilateral staged Magnetic Resonance Imaging-guided High-Intensity Focused Ultrasound thalamotomy is an effective and safe treatment for medication-refractory Essential Tremor, providing substantial tremor relief and improved quality of life with manageable side effects. These findings support its use as an alternative to more invasive neurosurgical procedures, especially in carefully selected patients.

## Teaser

Campins *et al*. found that bilateral staged focused ultrasound thalamotomy effectively reduces tremor and improves quality of life in essential tremor patients, with minimal side effects and a good safety profile, supporting its use as a treatment option.

## Graphical Abstract

**Graphical Abstract:**
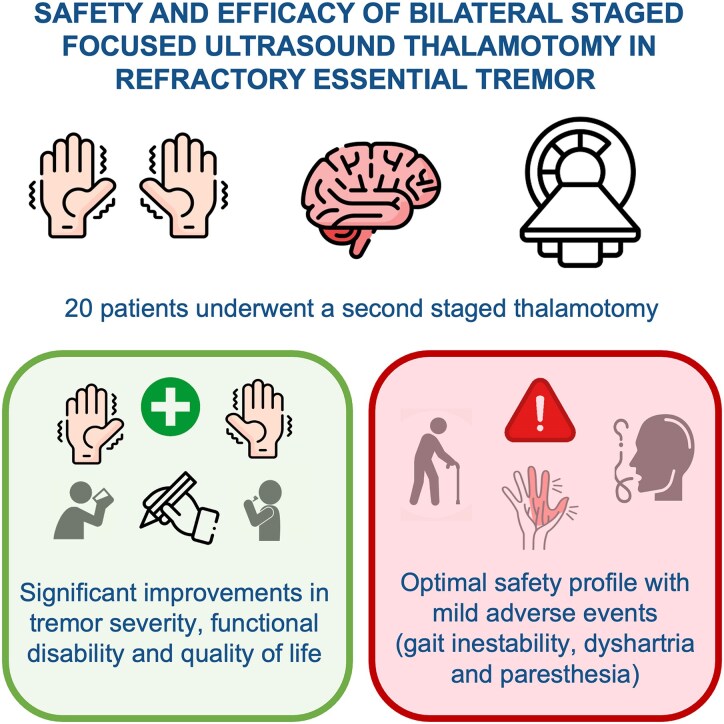

## Introduction

Essential Tremor (ET) is characterized by postural and/or kinetic tremor, affecting the upper limbs (most frequently bilaterally), head and voice.^1^ It affects approximately 0.9% of the adult population, with an increasing prevalence with age.^2^ Several pharmacological options are available; first-line treatment consists of propranolol and/or primidone, which achieves a 50% reduction in tremor severity in 70% of patients. Second-line therapies include topiramate, gabapentin and benzodiazepines.^3^ Patients who achieve insufficient benefit with medications can consider functional neurosurgical interventions. Indications for neurosurgical procedures include ineffectiveness or intolerance to oral treatments and/or contraindication to at least two oral treatments, one of which must be a first-line medication.

Currently, with the development of minimally invasive and incisionless procedures, interest in thalamotomies has re-emerged.^4,5^ Based on its demonstrated efficacy and safety, unilateral magnetic resonance image-guided high-intensity focused ultrasound (MRgFUS) thalamotomy gained approval in 2016 as a therapeutic intervention for refractory ET by regulatory agencies, including the U.S. Food and Drug Administration and the European Commission. Since then, this technique has become a widely adopted alternative to more invasive procedures such as DBS or radiofrequency lesions and has surpassed as well radiosurgery, a procedure, which lacks intraprocedural clinical monitoring with benefit and side effects often delayed.^6-8^ Typically, adverse events (AEs) are mild and commonly transient. Paresthaesia and ataxia stand out as the prevailing enduring issues.^5^ Preliminary safety and efficacy data of bilateral staged MRgFUS thalamotomy has led to its approval by Food and Drug Administration.^9,10^ However, these studies included a limited number of patients. Furthermore, there is limited evidence providing objective assessments of balance following bilateral MRgFUS thalamotomies.^11,12^

Here, we describe the results of our experience with bilateral staged MRgFUS thalamotomy for the treatment of medication-refractory ET. The primary objective was to evaluate whether staged bilateral thalamotomy can be performed with an acceptable safety profile and examining whether the benefits outweigh adverse effects. We emphasized the impact of this surgical treatment on balance and gait. The aim of this analysis is to provide insights for enhancing patient selection and optimizing surgical techniques.

## Materials and methods

### Patient population

From January 2022 to January 2024, consecutive patients who had been diagnosed with medication-refractory ET by a movement disorders specialist and had benefited from a previous MRgFUS unilateral thalamotomy at least 9 months before were enroled in this study.

Inclusion criteria were the following: age > 18 years, primary diagnosis of ET or ET plus confirmed by a movement disorders neurologist, successful completion of a first-side MRgFUS thalamotomy >9 months prior to enrolment, residual tremor on the untreated side negatively impacting patient quality of life despite the use of first-line antitremor medications (propranolol and/or primidone), a desire for second-side treatment and no clinically significant permanent AEs from the first thalamotomy. Patients were excluded if they had a history of stereotactic surgery or brain haemorrhage, were diagnosed with unstable cardiac or psychiatric disease, cognitive decline (assessed by formal neuropsychological evaluation), or had a skull density ratio ≤0.4 calculated from the CT screening scan. Exclusion criteria also applied to patients with clinically significant impairments in gait, balance, speech or swallowing, or those unable to comply with the 6 months follow-up schedule. Patients with asymmetric bilateral tremor who, after treatment of their dominant hand, had sufficiently improved their quality of life, or those who did not wish to undergo intervention on the other side due to concerns about the potential risk of developing side effects, were not included in this study. Additionally, some patients find the procedure on the first side challenging and prefer to avoid a similar experience.

### Statement of Ethics

This study received approval from the local Institutional Review Board, and informed consent was obtained from all participants as data collection started before Food and Drug Administration approval.

### Surgical procedure

All patients underwent a preoperative neuronavigation T1, T2 and diffusion-weighted imaging with a conventional 3 Tesla MRI scanner prior to surgery (Supplementary Table 4). Images were processed using a clinically approved stereotactic software platform (StealthStation S7 and S8 Medtronic). The target was the contralateral VIM nucleus corresponding to the upper limb requiring treatment. The presumptive site of the VIM nucleus was defined using indirect targeting coordinates: 14–15 mm laterally from midline or alternatively 11–11.5 mm from the wall of the III ventricle, 25% of the ACPC distance (anterior to the PC) and 3 mm above the depth of the intercommissural line. Tractography of the dento-rubro-cerebellothalamic tract confirmed and refined the final target coordinates, while tractography of the pyramidal tract and the lateral thalamic border ensured a safe margin from the internal capsule. Following insights from previous studies,^5^ the target for the second thalamotomy was adjusted 1 mm more superior (to 3 mm above the intercommisural line) than on the first side (2 mm above). This adjustment reflects our 5-year experience during which we have performed over 190 unilateral thalamotomies. We observed that perilesional oedema often extends caudally, contributing to side effects such as ataxia.^13^ To mitigate these risks, we established a safety margin by setting the target 1 mm superiorly during the second procedure.

Intraprocedural management did not differ from the first thalamotomy.^11^ The procedure began with scalp preparation and the fixation of a stereotactic frame (Exablate Neuro 4000, InSightec) under local anaesthesia. A flexible silicone membrane was placed around the head for the circulation of cooled (15°C–19°C) degassed water. The patient was positioned supine, and T2-weighted spin echo and FIESTA 3D MRI images were acquired using a 3 Tesla MRI scanner to localize the presumed VIM nucleus. An MRI-compatible phased-array transducer helmet was aligned and sonication commenced. Initial low-power sonications (10–20 s, 40°C–45°C peak temperatures) confirmed alignment. Power was gradually increased to achieve transient clinical effects at 50°C–53°C. Clinical monitoring was conducted throughout the procedure. Upon observing positive motor effects and no AEs, high-power sonication was performed to induce thermal ablation, targeting 55°C–60°C. Adjustments to target coordinates were made if side effects emerged or motor improvement was not observed. Once the first lesion was created, a second high-power sonication expanded and consolidated the ablation. In cases of insufficient temperature elevation, sonication duration was extended up to 50 s.

The final lesion was assessed on T2-weighted MRI obtained immediately after the procedure (Supplementary Table 4). All the interventions took place in an ambulatory setting.

### Outcome assessments

Three primary timepoints were defined: Baseline (before first thalamotomy), FUS1 (6 months after the first thalamotomy/serving as baseline for second thalamotomy) and FUS2 (6 months after the second thalamotomy). Clinical assessments were performed at baseline, FUS 1 and FUS2. The percentage of the patient’s self-reported improvement compared to Baseline was noted for each visit.

### Primary outcome

The primary efficacy outcome was defined as the change in the tremor score for the treated hand 6 months after the second thalamotomy (FUS2), compared with FUS1 and Baseline. Tremor on the treated side was scored using the Fahn–Tolosa–Marin Clinical Rating Scale for tremor (CRST, Clinical Rating Scale for Tremor) parts A + B that applied to the treated hand (range 0–32).^14^

### Secondary outcomes

Secondary outcomes included functional limitations in daily activities assessed by Part C of the CRST, European Quality of Life 5 Dimensions questionnaire (EQ-5D), the Quality of Life in Essential Tremor Questionnaire (QUEST) and observed AEs, particularly balance impairment. Functional disability was assessed using subsection Part C (range, 0–28) of the CRST, EQ-5D and the self-reported QUEST.^15^ Regarding the EQ-5D-5L scale, the severity index (ISEV) was calculated from the individual scores of the five dimensions. QUEST consists of 30 items divided into five dimensions (Communication, Work/Finances, Hobbies/Leisure, Physical and Psychosocial). The QUEST Summary Index (QUEST-SI) is calculated as the average of the 5 dimensions that make up the scale. Each item is scored from 0 to 4, resulting in a score range from 0 to 120. Higher scores imply lower quality of life associated with ET.

To elucidate the natural evolution of balance after the procedure, gait function was specifically assessed with the Berg Balance Scale (BBS), a 14-item objective measure that assesses static balance and fall risk in adults.^16^ Higher scores indicate better balance (scores from 41 to 56 indicate independent walking). A score of <45 indicates that individuals may be at greater risk of falling. For this analysis, a score <45 on the BBS 6 months after the treatment were considered a severe impact on patient’s balance and were rated as severe AE. A change in the BBS score of 4 points or greater was rated as a mild AE if it did not affect daily activities and as a moderate if it interfered with daily activities. A change of 3 or fewer was not considered clinically significant as it did not lead to a true change in balance.^17^ Baseline and 6 months neuropsychological assessments were also conducted.

Adverse events were collected at all assessments, including 1 month and 6 months after the second thalamotomy. AE were grouped in two categories:

1. Sonication-related: nausea, vomiting, dizziness and scalp burns.
2. Thalamotomy-related, further divided into five categories: sensory, motor, speech, balance and gait difficulties (subjective unsteadiness and objective ataxia) and dysmetria.

Thalamotomy-related AEs were further classified as: mild (minor inconvenience, not affecting routine daily activities), moderate (bothersome, interfering with routine daily activities) or severe (incapacitating, preventing performance of daily living activities).

### Statistical analysis

The descriptive analysis includes the most relevant statistics for continuous variables (mean, standard deviation, minimum, maximum, median and 25th and 75th percentiles) and categorical variables (absolute and relative frequencies). The normality of the variables and differences was assessed using the Shapiro–Wilk test, yielding diverse results. Regarding the CRST test, the subtotals scores (except voice tremor, head, speech scores and improvement percentage) conformed to a normal pattern. The global scores of the QUEST and EQ5D were also accepted as normal; however, the Berg scores were not. Therefore, given the sample size, the approach was parametric or non-parametric depending on the specific outcome analysed. The inferential analysis consisted of applying a general linear model ANOVA for repeated measures for each parameter that conformed to a normal distribution. Multiple comparisons between measurement times were performed using the Bonferroni test to adequately control the propagation of type I error. For non-normal parameters, a Friedman test was applied, with multiple Wilcoxon comparisons corrected for Bonferroni. The significance level used in the analyses was 5% (α=0.05). A Wilcoxon test achieves 80% power to detect changes consistent with a large effect size (*d* = 0.8) at a 95% confidence level. Statistical analyses were conducted with the use of SPSS software, version 15.0.

## Results

### Patients

Twenty patients (12 male), aged 66.50 ± 9.20 years, with long-standing ET (32.3 ± 17.50 years), received bilateral staged FUS thalamotomy and completed the 6 months follow-up. The mean time interval between treatments was 17.80 ± 7.6 months. Nineteen patients were right-handed, and all of them had been initially treated in the brain hemisphere contralateral to their dominant side. Demographics and surgical data from baseline and FUS1 (baseline for second thalamotomy) timepoints are shown in Table 1 and Supplementary Table 1, respectively.

**Table 1:** Demographics

		Baseline	FUS1
Sex, no. (%)			
Male	12 (60.00)		
Female	8 (40.00)		
Hand dominance, no. (%)			
Right-handed	19 (95.00)		
Left-handed	1 (5.00)		
Time between FUS1 and FUS2, months	17.80 ± 7.6		
Age at surgery, years		66.50 ± 9.20	66.73 ± 10.12
Disease duration, years		32.3 ± 17.50	33.64 ± 18.2
Thalamotomy side, no. (%)			
Left VIM		19 (95.00)	1 (5.00)
Right VIM		1 (5.00)	19 (95.00)
Skull density ratio – SDR		0.56 ± 0.10	0.57 ± 0.10

Continuous values are expressed in mean and standard deviation (SD). *n* = 20.

Timepoints: Baseline (before first thalamotomy) and FUS1 (6 months after the first thalamotomy/serving as baseline for second thalamotomy).

VIM, ventralis intermedius nucleus.

### Efficacy

**Figure 1:**
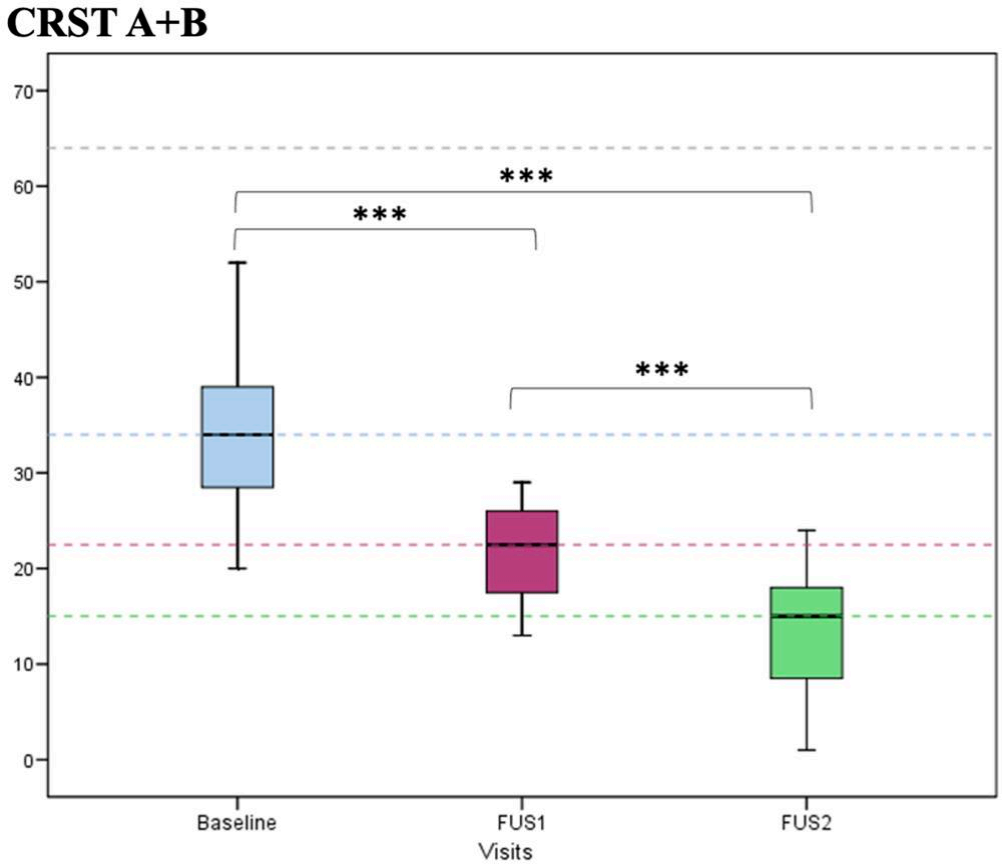
**CRST A + B CRST: clinical rating tremor scale.** CRST A + B at Baseline, FUS1 (6 months after the first MRgFUS) and FUS2 (6 months after the second MRgFUS). CRST A + B was calculated from the CRST Part A (3 items: resting, postural and action components of hand tremor) and Part B (5 tasks involving handwriting, drawing and pouring). *N* = 20. ANOVA *F* = 76.8; *P* < 0.001. Bonferroni´s *post hoc* test (all *P* < 0.001). ***P value < 0.001.

The mean total CRST score at Baseline was 56.60 ± 12.53 points (Table 2). Total CRST score decreased by 69.01% from Baseline to FUS2 (from 56.60 ± 12.53 to 18.15 ± 7.41, *P* < 0.001), with a 45.53% reduction at FUS1 (*P* < 0.001) and an additional decrease of 36.22% at FUS2 (*P* < 0.001). Tremor in the second-treated upper limb (CRST A + B score) improved by 53.25% at FUS2 (from 16.35 ± 3.12 at FUS1 to 7.70 ± 3.51 at FUS2, *P* < 0.001). By adding this benefit to that of the first ablation, bilateral upper limb CRST A + B decreased by 59.98% as compared with Baseline (from 33.55 ± 8.02 to 13.30 ± 6.80, *P* < 0.001; Fig. 1). Additionally, 100% and 93.3% of the patients self-reported marked improvement (≥75%) at FUS1 and FUS2, respectively, assessed by the subjective global evaluation of the CRST. Only one patient reported moderate improvement (25–49%) at FUS2. In the subgroup of patients presenting voice (*n* = 12) or head tremor (*n* = 12) at Baseline, only those affected by head tremor showed a significant improvement at FUS1 (*P* = 0.009). No additional improvement was observed after the second treatment. Voice scores remained stable from Baseline to the FUS2. Of those patients experiencing voice and head tremor, 75% and 83%, respectively, demonstrated improvements of at least one point in the CRST specific subscore at FUS2.

**Table 2:** Primary and secondary efficacy outcomes

	Baseline	FUS1	FUS2	Baseline-FUS1 *P* value	FUS1-FUS2 *P* value	Baseline-FUS2 *P* value
CRST Score						
Total CRST total (A + B + C) score	56.60 ± 12.53	29.70 ± 10.06	18.15 ± 7.41	<0.001*	<0.001*	<0.001*
CRST subscores						
CRST A subscore	19.20 ± 7.09	11.55 ± 4.37	6.65 ± 2.50	0.001*	<0.001*	<0.001*
CRST A voice total subscore^a^	1.00 (0.00–1.00)	0.00 (0.00–0.50)	0.00 (0.00–0.50)	0.249	1	0.099
CRST A head tremor total subscore^a^	2.00 (0.00–2.00)	0.00 (0.00–1.00)	0.00 (0.00–1.00)	0.009*	1	0.051
CRST B subscore	22.85 ± 5.70	14.85 ± 4.04	9.45 ± 5.46	<0.001*	<0.001*	<0.001*
Bilateral UL CRST (A + B) score	33.55 ± 8.02	21.85 ± 4.78	13.30 ± 6.80	<0.001*	<0.001*	<0.001*
First side UL CRST (A + B) score^b^	16.95 ± 5.50	5.50 ± 3.43	5.60 ± 4.60	<0.001*	0.053	<0.001*
Second side UL CRST (A + B) score^c^	16.60 ± 3.22	16.35 ± 3.12	7.70 ± 3.51	1	<0.001*	<0.001*
CRST C subscore	14.55 ± 4.14	3.30 ± 3.34	2.05 ± 2.09	<0.001*	0.308	<0.001*
CRST C speech subscore^a^	1.00 (0.00–2.00)	0.00 (0.00–2.00)	0.00 (0.00–1.00)	0.033*	1	0.014*

*n* = 20. *P value <0.05.

Continuous values are expressed in mean and standard deviation (SD).

Timepoints: Baseline, FUS1 (6 months after the first thalamotomy) and FUS 2 (6 months after the second thalamotomy).

^a^Non parametric distribution. Continuous values are expressed in median and interquartile range (IQR).

^b^Refers to the side treated in the first thalamotomy.

^c^Refers to the side treated in the second thalamotomy.

CRST, Clinical Rating Scale for Tremor; QUEST, Quality Of Life in Essential Tremor Questionnaire; UL, upper limb.

**Figure 2:**
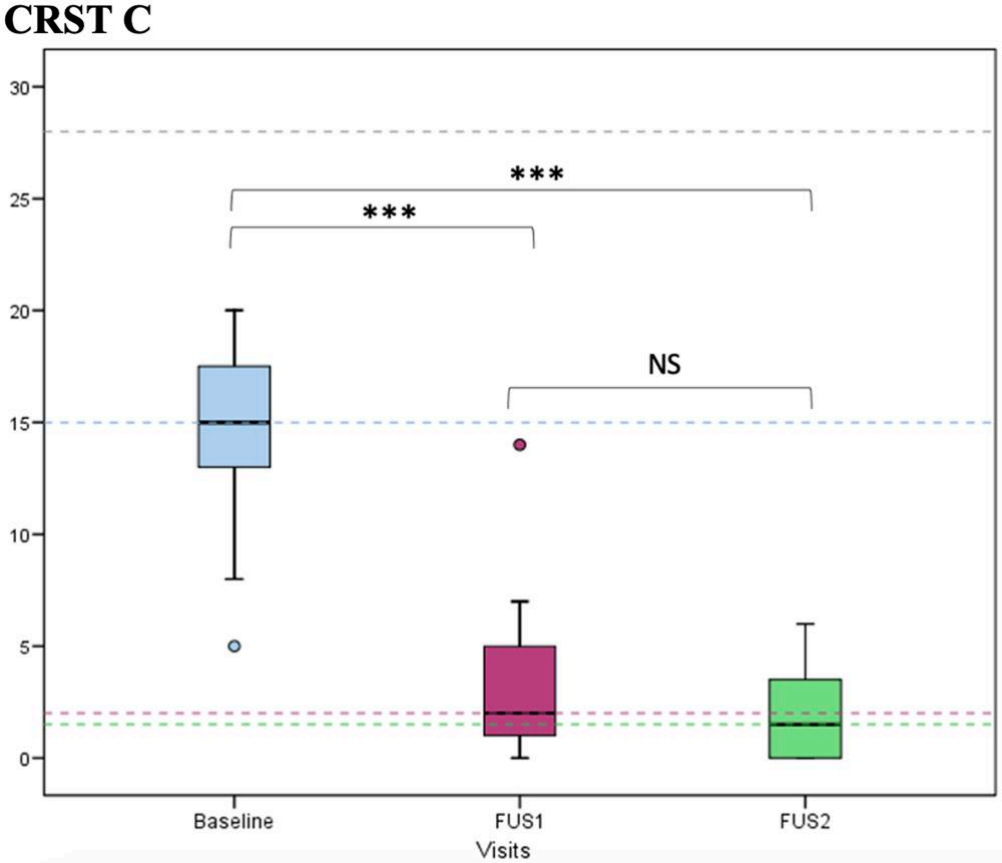
**CRST C. CRST: Clinical Rating Tremor Scale.** CRST C at Baseline, FUS1 (6 months after the first MRgFUS) and FUS2 (6 months after the second MRgFUS). *N* = 20. ANOVA *F* = 103.9; *P* < 0.001. Bonferroni´s *post hoc* test: Baseline versus FUS1 *P* < 0.001; FUS1 versus FUS2 *P* = 0.308; Baseline versus FUS2 *P* < 0.001. ***P value < 0.001. NS, no significance.

**Figure 3:**
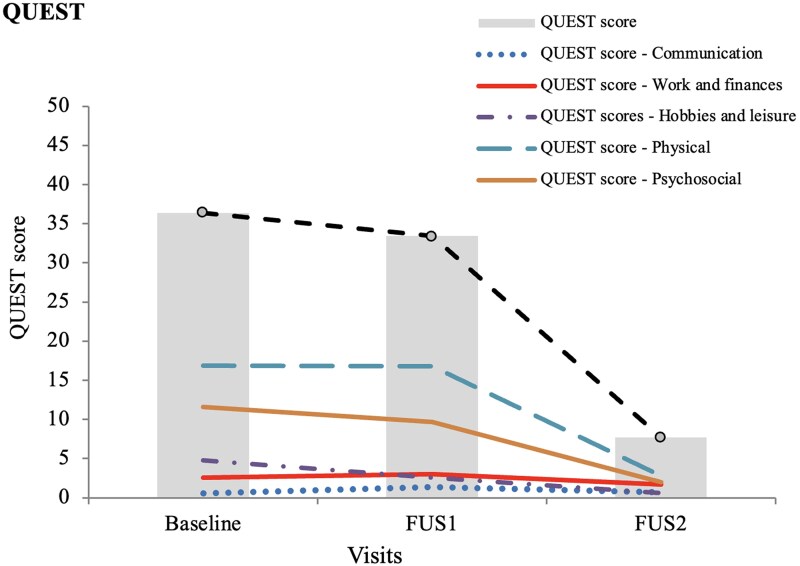
**QUEST. QUEST: Quality of Life in Essential Tremor Questionnaire (QUEST).** QUEST outcomes at Baseline, FUS1 (6 months after the first MRgFUS) and FUS2 (6 months after the second MRgFUS). *N* = 20. Overall score: ANOVA *F* = 16.9; *P* < 0.001. Bonferroni´s *post hoc* test: Baseline versus FUS1 *P* = 0.398; FUS1 versus FUS2 *P* = 0.005; Baseline versus FUS2 *P* < 0.001. Communication score: Friedman´s test Chi2 = 0.20; *P* = 0.905. Wilcoxon´s test corrected by Bonferroni: Baseline versus FUS1 *z* = 0 *P* = 1; FUS1 versus FUS2 *z* = 1.07 *P* = 0.855; Baseline versus FUS2 *z* = 0.27 *P* = 1. Work and finances score: Friedman´s test Chi2 = 6.74; *P* = 0.034. Wilcoxon´s test: Baseline versus FUS1 *z* = 0.74 *P* = 1; FUS1 versus FUS2 *z* = 1.56 *P* = 0.357; Baseline versus FUS2 *z* = 1.68 *P* = 0.276. Hobbies score: Friedman´s test Chi2 = 11.31; *P* = 0.003. Wilcoxon´s test: Baseline versus FUS1 *z* = 2.53 *P* = 0.033; FUS1 versus FUS2 z = 1.69 *P* = 0.273; Baseline versus FUS2 *z* = 1.99 *P* = 0.046. Physical score: *N* = 14, 20, 19. Friedman´s test Chi2 = 10.32; *P* = 0.006. Wilcoxon´s test: Baseline versus FUS1 *z* = 0.49 *P* = 1; FUS1 versus FUS2 *z* = 3.15 *P* = 0.006; Baseline versus FUS2 *z* = 3.11 *P* = 0.006. Psychosocial score: Friedman´s test Chi2 = 8.98; *P* = 0.011. Wilcoxon’s test: Baseline versus FUS1 *z* = 2.14 *P* = 0.099; FUS1 versus FUS2 *z* = 2.84 *P* = 0.012; Baseline versus FUS2 *z* = 2.08 *P* = 0.015.

There was an 84.91% improvement in disability (CRST C) from Baseline to FUS2 (from 14.55 ± 4.14 to 2.05 ± 2.09, *P* < 0.001), with 74.63% improvement at FUS1 (*P* < 0.001) and a further non-significant 41.35% reduction at FUS2 (from 3.30 ± 3.34 at FUS1 to 2.05 ± 2.09 at FUS2, *P* = 0.308; Fig. 2). Additionally, CRST C subitem 15 assessing tremor-related speech disability remained stable from Baseline to FUS2. Significant improvement of 75.64% in patients’ ratings of their quality of life, assessed using the QUEST (mean change in the total QUEST score at FUS2 compared with Baseline; *P* < 0.001) was confirmed. Notably, the hobbies/leisure, physical and psychosocial subdomains improved significantly during follow-up (*P* = 0.046, *P* = 0.0006, *P* = 0.015, respectively), while the work/finances and communication subdomains did not change significantly throughout the study (Supplementary Table 2 and Fig. 3).

After 6 months of the second thalamotomy (FUS2), only one patient experienced a reduction of 4 points or more in the BBS score compared to Baseline. This worsening in balance was rated as mild, as it did not affect daily activities. Objective ataxia was therefore mild, and no patients showed a BBS score of <45 points (higher risk of falls and walking with assistance). Notably, mild subjective unsteadiness was frequent after ultrasound thalamotomy, but balance scores remained stable throughout the study, with no significant differences at any timepoint.

### Safety

Adverse events after second thalamotomy were all mild (Table 3 and Supplementary Table 3). One and three out of 20 patients developed subjective gait instability (catalogued as mild tandem ataxia by explorers) after 6 months follow-up of the first (FUS1) and second thalamotomy (FUS2), respectively. Nevertheless, no instances of moderate to severe objective ataxia were noticed in any patients. Two patients showed dysarthria (rated as mild) 1 month after the second thalamotomy, which persisted in 1 at 6 months follow-up visit. Only one patient presented with persistent mild finger paresthaesia at FUS1. At FUS2, 5 additional patients presented paresthaesia, in all cases involving the tongue. Facial asymmetry was detected in one patient 1 month after the second procedure and was resolved at 6 months visit (FUS2). No limb weakness was observed after any procedure. Intraprocedural AEs did not differ from those reported for unilateral treatments and were all mild and transient.

**Table 3:** Thalamotomy-related adverse events (at 6 months)—no. of patients (%)

	First thalamotomy			Second thalamotomy		
	1 month	3 months	6 months	1 month	3 months	6 months
Dysarthria	1 (5)	0 (0)	0 (0)	2 (10)	2 (10)	1 (5)
Facial asymmetry	0 (0)	0 (0)	0 (0)	1 (0)	0 (0)	0 (0)
Limb weakness	0 (0)	0 (0)	0 (0)	0 (0)	0 (0)	0 (0)
Paresthaesia	2 (10)	1 (5)	1 (5)	9 (45)	7 (35)	6 (30)
Dysmetria	1 (5)	0 (0)	0 (0)	1 (5)	0 (0)	1 (5)
Subjective gait disturbance^a^	5 (25)	1 (5)	1 (5)	4 (20)	3 (15)	3 (15)
Objective gait disturbance	1 (5)	1 (5)	0 (0)	3 (15)	3 (15)	3 (15)
Total adverse events, according to severity 8 at 6 months), no. of events						
Mild	10	3	2	20	15	14
Moderate	0	0	0	0	0	0
Severe	0	0	0	0	0	0

Thalamotomy-related adverse events. *n* = 20.

At 6 months, no. of patients (%).

^a^Patients who reported ‘unsteady gait’ were those who reported having less equilibrium while walking.

Complete neuropsychological testing before and 6 months after the second thalamotomy can be found in the online appendix (Supplementary Table 5). No differences were detected between evaluations.

## Discussion

In the early years of movement disorder surgery, thalamic lesioning techniques were widely explored. However, during the 1960s, up to 60% of patients undergoing simultaneous or staged bilateral thalamotomies for Essential Tremor, Parkinson’s disease, or dystonia experienced persistent complications, primarily speech and gait disturbances.^18^ These adverse effects were particularly common in patients with pre-existing impairments or worsening symptoms after an initial unilateral procedure. The highest complication rates occurred before the advent of MRI and modern stereotactic techniques, leading to concerns about the safety of staged bilateral lesions and ultimately driving the shift toward deep brain stimulation (DBS) as the preferred surgical approach.^19,20^ Since the early 21st century, DBS has been the standard intervention for movement disorders, demonstrating superior efficacy over best medical management.^21-24^ Its adjustable stimulation allows for a more refined balance between symptom relief and adverse effects compared to permanent lesioning. However, DBS is associated with gradual tolerance to its effects^25^ and, though infrequent, significant complications, including perioperative risks (bleeding, infection and neurological deficits) and long-term issues such as device malfunction, lead fracture, or skin erosion. Additionally, bilateral thalamic stimulation carries a notable risk of dysarthria (22–75%) and gait disturbances (56–85.7%), sometimes requiring stimulation adjustments or discontinuation to mitigate side effects.^26,27^

The feasibility of performing a second-staged MRgFUS thalamotomy for Essential Tremor represents a relatively recent advancement. Publications supporting the efficacy and safety of bilateral MRgFUS procedures remain limited, primarily derived from small patient cohorts,^9,10,12,28^ with only a few larger, recent series offering additional insights.^29^ Although ET is typically a bilateral condition, significant relief of tremor and improvements in activities of daily living can often be achieved through unilateral procedures. As a result, not all patients with bilateral symptoms seek or qualify for a second procedure. In our series, second procedures were offered exclusively to patients whose tremor in the second hand remained moderate and interfered with daily activities despite successful unilateral MRgFUS thalamotomy. Patients with pre-existing balance or gait issues, dysarthria, dysphagia or cognitive decline were excluded from consideration for a second intervention.

Our findings align with previous studies, demonstrating a 64% improvement in hand tremor on the first treated side at 6 months. Improvement after the second procedure, while still significant at 53.25%, was slightly lower. This discrepancy may reflect a more conservative approach during the second procedure or intentional modifications in targeting, such as planning a more rostral lesion. Despite the relatively short 6 months follow-up period, previous research suggests that benefits observed at this time point are generally sustained over longer periods.^30,31^

Secondary outcomes, including reduced functional limitations in daily activities and improved self-reported quality of life, were also substantial. Disability scores (CRST C) demonstrated a sustained 74.63% improvement from baseline after the second MRgFUS procedure (Fig. 2). Notably, certain quality of life domains, such as physical and psychological well-being and leisure activities, showed greater improvement following the second thalamotomy. However, the small sample size may account for the lack of statistical significance across all quality of life dimensions, contrasting with findings from larger studies demonstrating significant improvements in multiple quality of life areas.^11^ Domains such as work/financial and communication showed minimal changes, likely due to the retired status of most participants and the mild baseline impact of voice or head tremor.

The impact of bilateral MRgFUS thalamotomy on axial tremor remains a subject of debate. While some studies report improvements in voice and head tremor,^10,29^ our findings revealed that 75% and 83% of patients experienced at least one point improvement in voice and head tremor, respectively. However, a statistically significant reduction was observed only in head tremor, potentially due to the mild baseline severity of axial tremor in our cohort. Axial tremor management poses unique challenges. Evidence regarding its improvement with DBS remains sparse.^32,33^ Despite the established efficacy of DBS for appendicular tremor, its application to axial symptoms has shown inconsistent results. Targeting more medial areas within the VIM to address axial symptoms increases the risk of side effects, such as dysarthria, limiting the practicality of such approach.^34^ Moreover, the feasibility of staged bilateral thalamotomy depends heavily on the absence of axial side effects following the initial procedure. Consequently, neither the emerging approach of staged bilateral MRgFUS nor the established DBS can yet be considered fully reliable for treating axial tremors.

Bilateral staged thalamotomy may pose a greater risk compared to unilateral procedures due to the involvement of homologous brain structures on both sides. In our study, mild AEs were reported in 70% of patients at 6 months, consistent with previous findings. Importantly, no moderate-to-severe adverse effects occurred following the second thalamotomy. Mild dysarthria was observed in 5% of patients, a common side effect associated with thalamic lesions. The incidence of speech difficulties after thalamotomy has historically been higher, especially in bilateral procedures.^18,33,34^ Dysarthria following bilateral staged thalamotomy can result from lesion extension affecting various structures. Excessively lateral lesions may involve the corticobulbar tract, while medial VIM involvement—containing the homuncular representation of the face, tongue and larynx—can also contribute. Inferior lesion extension encroaching on the rubrocerebello-thalamic tract may further lead to cerebellar symptoms such as dysmetria or ataxia. Notably, evidence from thalamic DBS suggests that medial thalamic stimulation exacerbates speech deterioration, underscoring the critical importance of precise targeting.^13^ In our study, the mild nature of dysarthria suggests that modern stereotactic techniques, with more precise targeting, smaller lesion sizes and careful patient selection, have reduced the risk compared to earlier approaches. Similarly, gait problems may result from different neurological deficits: weakness when the corticospinal tract is affected, ataxia or lower limb dysmetria when lesions involve the rubrocerebello-thalamic tract.^13^ In our series, gait disturbances were reported in 30% of patients (15% subjective and 15% objective), with none requiring walking aids. This aligns with previous reports on thalamic lesions, where gait difficulties were commonly observed, particularly in bilateral interventions. Interestingly, no significant differences were observed when comparing patients by age or baseline BBS scores, although older patients with lower baseline BBS scores may have an increased risk of developing gait disturbances, especially in bilateral interventions.^17^ This highlights the need for further research to better characterize these risks and refine selection criteria. Paresthaesia was more frequent after the second lesion (30% versus 5%), possibly due to the asymmetrical targeting strategy employed, with the second lesion placed more superiorly. While previous studies using asymmetrical targeting did not report higher rates of dysesthesia,^9,28^ the different targeting approach (with more superior and anterior placements for the second lesion) may have contributed to these findings. The anatomical proximity of the medial lemniscus, the ventral caudal nucleus and its afferent connections play a crucial role in the occurrence of sensory disturbances. Cognitive decline has historically been a concern with bilateral ablative techniques. However, in our study, no cognitive impairments were identified during neuropsychological assessments at 6 months, consistent with other reports on bilateral MRgFUS.^10,12,35^ This contrasts with the variable cognitive outcomes observed with unilateral Gamma Knife^36-38^ and bilateral staged RF thalamotomy,^39^ which remain incompletely assessed. In Parkinson’s disease, cognitive changes related to STN DBS have often been attributed to stimulation outside the STN or lesions caused by electrode trajectories. For instance, reduced verbal fluency has been linked to more anterior cortical entry points, while executive dysfunction has been associated with electrode trajectories through the caudate nucleus.^40,41^ The same trajectory-related risks may apply to DBS targeting the VIM, which often requires narrower entry angles. However, it is important to note that much of the evidence in this context stems from Parkinson's disease populations, who inherently face a higher risk for cognitive decline compared to patients with Essential Tremor.

Despite these improvements, early complications of bilateral lesion surgery led to a preference for DBS due to its reversibility.^19^ However, DBS itself presents challenges, including high rates of dysarthria (22–75%),^6,27,34^ ataxia (56–85.7%)^32^ and dysphagia (18.8%).^34^ Neurostimulation-related side effects may necessitate alternating stimulation settings or even preclude bilateral stimulation entirely. Some centres opt for unilateral DBS, particularly for older patients, or revert to unilateral RF thalamotomy due to its more favourable cost-effectiveness ratio. Habituation to the effects of DBS is a critical factor to consider. Long-term studies with 6-year follow ups indicate that tremor worsens at a rate of 0.37 points per month.^25^ Of this deterioration, 13% is attributed to habituation to DBS, while 87% is driven by disease progression. However, only few studies have explored this phenomenon in the context of MRgFUS.^4^ This raises an important question: Is the deterioration rate following thalamotomy exclusively explained by disease’s progression or is there also an intrinsic habituation to the effect of thermolesion?

The issue of symmetry in targeting for the second lesion remains debated. Symmetrical targeting simplifies planning but may increase the risk of axial side effects by involving structures symmetrically linked to axial symptoms.^10,13^ Asymmetrical targeting, such as the rostral placement of the second lesion used in our series, offers an alternative to mitigate these risks. Specifically, we planned the second lesion 1 mm superiorly (*z* = 3 mm above ACPC) to minimize the risk of gait disturbances while maintaining efficacy.^28,42^ Considering the more modest reduction in benefit following the second thalamotomy (53.25% versus 64%) and the absence of a significantly improved AE profile, the debate regarding symmetric versus asymmetric targeting in this technique remains unresolved. The existing evidence is insufficient to draw definitive conclusions, emphasizing the need for rigorous, systematic investigations to comprehensively assess both the efficacy and safety of this approach.

Since the Food and Drug Administration approved MRgFUS in 2016, there has been a notable shift from DBS to MRgFUS for unilateral treatments, and our experience indicates a similar trend for bilateral procedures. Recently, simultaneous bilateral MRgFUS VIM thalamotomy has been proposed as a viable alternative, with preliminary findings indicating safety and efficacy.^43^ However, given the limited cumulative experience with bilateral procedures, staged approaches remain preferable, particularly since intraoperative monitoring of balance—crucial for minimizing side effects—is not feasible during a single session.

This study is limited by its small sample size and relatively short follow-up period, restricting our ability to evaluate long-term outcomes and safety comprehensively. Larger studies with extended follow-up are essential to assess the sustainability of benefits, the resolution of adverse effects and the optimization of targeting strategies for bilateral staged MRgFUS.

Staged bilateral MRgFUS thalamotomy appears to be a promising option for carefully selected patients with refractory bilateral Essential Tremor. The procedure achieves significant tremor relief with a favourable safety profile. However, careful patient selection is critical, taking into account individual characteristics, comorbidities and the potential impact of bilateral interventions. Unilateral procedures often provide sufficient symptom control, underscoring the importance of evaluating the need for bilateral treatment on a case-by-case basis. Establishing a standardized assessment of side effects will enhance comparisons of different targeting approaches and guide clinical decision-making. Future research should focus on larger, multicentre studies and long-term follow-up to refine targeting strategies and evaluate durability. Advances in MRgFUS have revitalized interest in surgical treatments for tremor, offering a non-invasive alternative to traditional approaches. Integrating these advancements with evidence from other ablative procedures will help maximize therapeutic outcomes and expand the applicability of MRgFUS in clinical practice.

## Supplementary Material

fcaf168_Supplementary_Data

## Data Availability

Data are fully available. Please contact the corresponding author.

## References

[1] Sullivan KL . NDT-4795-overview-of-essential-tremor. Neuropsychiatr Dis Treat. 2010;6(1):401–408. 20856604 10.2147/ndt.s4795 PMC2938289

[2] Song P, Zhang Y, Zha M, et al The global prevalence of essential tremor, with emphasis on age and sex: A meta-analysis. J Glob Health. 2021;11:1–8. 10.7189/jogh.11.04028 PMC8035980 33880180

[3] Zesiewicz TA, Elble RJ, Louis ED, et al Evidence-based guideline update: Treatment of essential tremor: Report of the quality standards subcommittee of the American academy of neurology. Neurology. 2011;77(19):1752–1755. 22013182 10.1212/WNL.0b013e318236f0fd PMC3208950

[4] Miller WK, Becker KN, Caras AJ, et al Magnetic resonance-guided focused ultrasound treatment for essential tremor shows sustained efficacy: A meta-analysis. Neurosurg Rev. 2022;45(1):533–544. 33978922 10.1007/s10143-021-01562-w

[5] Elias WJ, Lipsman N, Ondo WG, et al A randomized trial of focused ultrasound thalamotomy for essential tremor. N Engl J Med. 2016;375(8):730–739. 27557301 10.1056/NEJMoa1600159

[6] Giordano M, Caccavella VM, Zaed I, et al Comparison between deep brain stimulation and magnetic resonance-guided focused ultrasound in the treatment of essential tremor: A systematic review and pooled analysis of functional outcomes. J Neurol Neurosurg Psychiatry. 2020;91(12):1270–1278. 33055140 10.1136/jnnp-2020-323216

[7] Altinel Y, Alkhalfan F, Qiao N, Velimirovic M. Outcomes in lesion surgery versus deep brain stimulation in patients with tremor: A systematic review and meta-analysis. World Neurosurg. 2019;123:443–452.e8. 30500587 10.1016/j.wneu.2018.11.175

[8] Higuchi Y, Matsuda S, Serizawa T. Gamma knife radiosurgery in movement disorders: Indications and limitations. Mov Disord. 2017;32(1):28–35. 27029223 10.1002/mds.26625

[9] Iorio-Morin C, Yamamoto K, Sarica C, et al Bilateral focused ultrasound thalamotomy for essential tremor (BEST-FUS phase 2 trial). Mov Disord. 2021;36(11):2653–2662. 34288097 10.1002/mds.28716

[10] Martínez-Fernández R, Mahendran S, Pineda-Pardo JA, et al Bilateral staged magnetic resonance-guided focused ultrasound thalamotomy for the treatment of essential tremor: A case series study. J Neurol Neurosurg Psychiatry. 2021;92(9):927–931. 33906933 10.1136/jnnp-2020-325278

[11] Sastre-Bataller I, Campins-Romeu M, Marcos-Carrión A, et al Gait function after high-intensity focused ultrasound thalamotomy for essential tremor: Searching for technique optimization. Stereotact Funct Neurosurg. 2023;101(1):12–21. 36696885 10.1159/000527374

[12] Scantlebury N, Rohringer CR, Rabin JS, et al Safety of bilateral staged magnetic resonance-guided focused ultrasound thalamotomy for essential tremor. Mov Disord Clin Pract. 2023;10(10):1559–1561. 37868927 10.1002/mdc3.13882 PMC10585969

[13] Boutet A, Ranjan M, Zhong J, et al Focused ultrasound thalamotomy location determines clinical benefits in patients with essential tremor. Brain. 2018;141(12):3405–3414. 30452554 10.1093/brain/awy278

[14] Stacy MA, Elble RJ, Ondo WG, Wu SC, Hulihan J. Assessment of interrater and intrarater reliability of the fahn-tolosa-marin tremor rating scale in essential tremor. Mov Disord. 2007;22(6):833–838. 17343274 10.1002/mds.21412

[15] Tröster AI, Pahwa R, Fields JA, Tanner CM, Lyons KE. Quality of life in essential tremor questionnaire (QUEST): Development and initial validation. Parkinsonism Relat Disord. 2005;11(6):367–373. 16103000 10.1016/j.parkreldis.2005.05.009

[16] Berg K, Wood-Dauphinee S, Williams JI, Gayton D. Measuring balance in the elderly: Preliminary development of an instrument. Physiother Can. 1989;41(6):304–311.

[17] Donoghue D, Murphy A, Jennings A, et al How much change is true change? The minimum detectable change of the berg balance scale in elderly people. J Rehabil Med. 2009;41(5):343–346. 19363567 10.2340/16501977-0337

[18] Alshaikh J, Fishman PS. Revisiting bilateral thalamotomy for tremor. Clin Neurol Neurosurg. 2017;158(March):103–107. 28505539 10.1016/j.clineuro.2017.04.025

[19] Tasker RR . Deep brain stimulation is preferable to thalamotomy for tremor suppression. Surg Neurol. 1998;49(2):145–153. 9457264 10.1016/s0090-3019(97)00459-x

[20] Schuurman PR, Bosch DA, Bossuyt PM, Bonsel GJ, van Someren EJ, de Bie RM, Merkus MP, Speelman JD. A comparison of continuous thalamic stimulation and thalamotomy for suppression of severe tremor. N Engl J Med. 2000;342 (7):461–468. doi: 10.1056/NEJM200002173420703 10675426

[21] Taha JM, Janszen MA, Favre J. Thalamic deep brain stimulation for the treatment of head, voice, and bilateral limb tremor. J Neurosurg. 1999;91(1):68–72. 10389882 10.3171/jns.1999.91.1.0068

[22] Ferreira JJ, Mestre TA, Lyons KE, et al MDS evidence-based review of treatments for essential tremor. Mov Disord. 2019;34(7):950–958. 31046186 10.1002/mds.27700

[23] Okun MS . Deep-brain stimulation for Parkinson’s disease. N Engl J Med. 2006;355(21):2256–2256. 17124028 10.1056/NEJMc062545

[24] Magown P, Andrade RA, Soroceanu A, Kiss ZHT. Deep brain stimulation parameters for dystonia: A systematic review. Parkinsonism Relat Disord. 2018;54:9–16. 29705556 10.1016/j.parkreldis.2018.04.017

[25] Paschen S, Forstenpointner J, Becktepe J, et al Long-term efficacy of deep brain stimulation for essential tremor: An observer-blinded study. Neurology. 2019;92(12):E1378–E1386. 30787161 10.1212/WNL.0000000000007134

[26] Wong JK, Hess CW, Almeida L, et al Deep brain stimulation in essential tremor: Targets, technology, and a comprehensive review of clinical outcomes. Expert Rev Neurother. 2020;20(4):319–331. 32116065 10.1080/14737175.2020.1737017 PMC7174089

[27] Ferreira Felloni Borges Y, Cheyuo C, Lozano AM, Fasano A. Essential tremor–deep brain stimulation vs. Focused ultrasound. Expert Rev Neurother. 2023;23(7):603–619. 37288812 10.1080/14737175.2023.2221789

[28] Fukutome K, Hirabayashi H, Osakada Y, Kuga Y, Ohnishi H. Bilateral magnetic resonance imaging-guided focused ultrasound thalamotomy for essential tremor. Stereotact Funct Neurosurg. 2022;100(1):44–52. 34515233 10.1159/000518662

[29] Kaplitt MG, Krishna V, Eisenberg HM, et al Safety and efficacy of staged, bilateral focused ultrasound thalamotomy in essential tremor an open-label clinical trial. JAMA Neurol. 2024;81(9):939–946. 39073822 10.1001/jamaneurol.2024.2295 PMC11287440

[30] Park YS, Jung NY, Na YC, Chang JW. Four-year follow-up results of magnetic resonance-guided focused ultrasound thalamotomy for essential tremor. Mov Disord. 2019;34(5):727–734. 30759322 10.1002/mds.27637

[31] Cosgrove GR, Lipsman N, Lozano AM, et al Magnetic resonance imaging–guided focused ultrasound thalamotomy for essential tremor: 5-year follow-up results. J Neurosurg. 2022;0:1–6. 10.3171/2022.6.JNS212483 PMC10193464 35932269

[32] Kleiner-Fisman G, Herzog J, Fisman DN, et al Subthalamic nucleus deep brain stimulation: Summary and meta-analysis of outcomes. Mov Disord. 2006;21(SUPPL. 14):290–304. 10.1002/mds.20962 16892449

[33] Dallapiazza RF, Lee DJ, De Vloo P, et al Outcomes from stereotactic surgery for essential tremor. J Neurol Neurosurg Psychiatry. 2019;90(4):474–482. 30337440 10.1136/jnnp-2018-318240 PMC6581115

[34] Alomar S, King NKK, Tam J, Bari AA, Hamani C, Lozano AM. Speech and language adverse effects after thalamotomy and deep brain stimulation in patients with movement disorders: A meta-analysis. Mov Disord. 2017;32(1):53–63. 28124434 10.1002/mds.26924

[35] Bruno F, Catalucci A, Varrassi M, et al Bilateral MRgFUS thalamotomy for tremor: A safe solution? Case report and review of current insights. Clin Neurol Neurosurg. 2020;197(August):106164. 32911249 10.1016/j.clineuro.2020.106164

[36] Prajakta G, Horisawa S, Kawamata T, Taira T. Feasibility of staged bilateral radiofrequency ventral intermediate nucleus thalamotomy for bilateral essential tremor. World Neurosurg. 2019;125:e992–e997. 30771542 10.1016/j.wneu.2019.01.224

[37] Hugdahl K, Wester K. Neurocognitive correlates of stereotactic thalamotomy and thalamic stimulation in parkinsonian patients. Brain Cogn. 2000;42(2):231–252. 10744922 10.1006/brcg.1999.1102

[38] Nijhawan SR, Banks SJL, Aziz TZ, et al Changes in cognition and health-related quality of life with unilateral thalamotomy for parkinsonian tremor. J Clin Neurosci. 2009;16(1):44–50. 19019683 10.1016/j.jocn.2008.03.008

[39] Horisawa S, Nonaka T, Kohara K, Mochizuki T, Kawamata T, Taira T. Bilateral radiofrequency ventral intermediate thalamotomy for essential tremor. Stereotact Funct Neurosurg. 2023;101(1):30–40. 36720205 10.1159/000528825

[40] Witt K, Granert O, Daniels C, et al Relation of lead trajectory and electrode position to neuropsychological outcomes of subthalamic neurostimulation in Parkinson’s disease: Results from a randomized trial. Brain. 2013;136(7):2109–2119. 23801735 10.1093/brain/awt151

[41] Le Goff F, Derrey S, Lefaucheur R, et al Decline in verbal fluency after subthalamic nucleus deep brain stimulation in Parkinson’s disease: A microlesion effect of the electrode trajectory? J Parkinsons Dis. 2015;5(1):95–104. 25374271 10.3233/JPD-140443

[42] Gallay MN, Moser D, Rossi F, et al MRgFUS pallidothalamic tractotomy for chronic therapy-resistant Parkinson’s disease in 51 consecutive patients: Single center experience. Front Surg. 2020;6(January):1–13. 10.3389/fsurg.2019.00076 PMC6971056 31993437

[43] Nabiullina DI, Galimova RM, Illarioshkin SN, et al Experience of staged and simultaneous bilateral thalamotomy using MR-guided focused ultrasound in the treatment of essential tremor. Zhurnal Nevrologii i Psihiatrii imeni SS Korsakova. 2023;123(7):65–73. 10.17116/jnevro202312307165 37490667

