# Supplementary material for: Safety and efficacy of bilateral staged focused ultrasound thalamotomy in refractory essential tremor
Source: Brain Commun. 2025 May 2;7(3):fcaf168. doi: 10.1093/braincomms/fcaf168 (PMC12065003; doi:10.1093/braincomms/fcaf168)
Supplement: fcaf168_Supplementary_Data [file fcaf168_supplementary_data.docx]

**Safety and efficacy of bilateral staged focused ultrasound thalamotomy in refractory essential tremor**

Marina Campins-Romeu, MD; Rebeca Conde-Sardón MD, PhD; Isabel Sastre-Bataller, MD; Raquel Baviera-Muñoz, MD, PhD; Mireya Losada-López, MD; Carlos Morata-Martínez, MD; María José Ibáñez-Juliá, MD; José Luís León-Guijarro, MD, PhD; Julia Pérez-García; Luis Raga-Rodríguez; Andrés M Lozano, MD, PhD; Antonio Gutiérrez-Martín, MD; PhD; Irene Martínez-Torres, MD, PhD.

**SUPPLEMENTARY MATERIAL**

**SUPPLEMENTARY TABLES**

| **Supplementary Table 1. Surgical data** | |  |  |
| --- | --- | --- | --- |
|  |  | **Baseline** | **FUS1** |
| Thalamotomy side - no, (%) |  |  |  |
| *Left VIM* |  | 19 (95.00) | 1 (5.00) |
| *Right VIM* |  | 1 (5.00) | 19 (95.00) |
| Skull Density Ratio - SDR |  | 0.56 ± 0.10 | 0.57 ± 0.10 |
| Baseline M-L coordinate^a^ |  | 14.07 ± 1.06 | 14.03 ± 1.16 |
| Baseline A-P coordinate^b^ |  | 6.03 ± 0.59 | 5.67 ± 0.65 |
| Baseline S-I coordinate^c^ |  | 2.01 ± 0.26 | 2.90 ± 0.15 |
| Sonications - no |  | 6.60 ± 2.67 | 5.20 ± 1.11 |
| Thermoablative sonications^d^ - no |  | 1.86 ± 0.95 | 1.93 ± 0.70 |
| Coordinates adjustments^e^- no |  | 0.68 ± 0.67 | 0.80 ± 0.89 |
| Sonication duration - min |  | 31.05 ± 24.28 | 25.40 ± 15.64 |
| Final Energy^f^ - Jules |  | 16639.60 ± 13659.30 | 17445.25 ± 15080.27 |
| Final Power^f^ - Watts |  | 764.75 ± 97.39 | 796.80 ± 142.95 |
| Final Average Temperature^f^ - ºC |  | 57.05 ± 3.66 | 56.45 ± 2.33 |
| Final Maximum Temperature^f^ - ºC |  | 63.11 ± 5.41 | 60.60 ± 3.55 |
| Immediate post-treatment M-L coordinate^a^ |  | 14.13 ± 1.15 | 14.22 ± 1.43 |
| Immediate post-treatment A-P coordinate^b^ |  | 5.55 ± 0.77 | 5.65 ± 1.03 |
| Immediate post-treatment S-I coordinate^c^ |  | 1.85 ± 0.35 | 2.44 ± 0.48 |

**Supplementary Table 1. Surgical data**

Continuous values are expressed in mean and standard deviation (SD). n=20.

Timepoints: Baseline (before first thalamotomy) and FUS1 (6 months after the first thalamotomy/serving as baseline for second thalamotomy).

VIM ventralis intermedius nucleus.

^a^M-L indicates Medial-Lateral.

^b^A-P indicates Anterior-Posterior.

^c^S-I indicates Supero-Inferior.

^d^Refers to the number of sonications that met mean target temperatures between 55°C and 60°C.

^e^Refers to the number of adjustments of the target coordinates if intraprocedural involuntary movements or adverse events were encountered.

^f^Refers to those values applied at the last sonication.

| **Supplementary Table 2. Primary and Secondary Efficacy Outcomes** | | | | | | | | | |
| --- | --- | --- | --- | --- | --- | --- | --- | --- | --- |
|  | **Baseline** | **FUS1** | **FUS2** | **Baseline-FUS1 improvement**  **(%)** | **FUS1-FUS2 improvement**  **(%)** | **Baseline-FUS2 improvement**  **(%)** | **Baseline-FUS1**  **Pvalue** | **FUS1-FUS2**  **Pvalue** | **Baseline-FUS2**  **Pvalue** |
| CRST Score |  |  |  |  |  |  |  |  |  |
| Total CRST total (A+B+C) score | 56.60 ± 12.53 | 29.70 ± 10.06 | 18.15 ± 7.41 | 45.53 ± 20.18 | 36.22 ± 27.52 | 69.01 ± 13.22 | <0.001* | <0.001* | <0.001* |
| CRST subscores |  |  |  |  |  |  |  |  |  |
| CRST A subscore | 19.20 ± 7.09 | 11.55 ± 4.37 | 6.65 ± 2.50 | 33.33 ± 31.88 | 35.23 ± 30.80 | 60.73 ± 21.68 | 0.001* | <0.001* | <0.001* |
| CRST A voice total subscore* | 1.00 (0.00-1.00) | 0.00 (0.00-0.50) | 0.00 (0.00-0.50) | 100.00 (0.00-100.00) | 50.00 (0.00-100.00) | 100.00 (25.00-100.00) | 0.249 | 1 | 0.099 |
| CRST A head tremor total subscore* | 2.00 (0.00-2.00) | 0.00 (0.00-1.00) | 0.00 (0.00-1.00) | 50.00 (50.00-100.00) | 75.00 (50.00-100.00) | 75.00 (50.00-100.00) | 0.009* | 1 | 0.051 |
| CRST B subscore | 22.85 ± 5.70 | 14.85 ± 4.04 | 9.45 ± 5.46 | 32.10 ± 21.84 | 39.86 ± 31.30 | 59.02 ± 20.35 | <0.001* | <0.001* | <0.001* |
| Bilateral UL CRST (A+B) score | 33.55 ± 8.02 | 21.85 ± 4.78 | 13.30 ± 6.80 | 31.97 ± 19.19 | 41.36 ± 27.26 | 59.98 ± 18.31 | <0.001* | <0.001* | <0.001* |
| First side UL CRST (A+B) score^a^ | 16.95 ± 5.50 | 5.50 ± 3.43 | 5.60 ± 4.60 | 64.00 ± 26.78 | 2.41 ± 78.44 | 69.16 ± 23.64 | <0.001* | 0.053 | <0.001* |
| Second side UL CRST (A+B) score^b^ | 16.60 ± 3.22 | 16.35 ± 3.12 | 7.70 ± 3.51 | 0.16 ± 16.33 | 53.25 ± 19.83 | 52.60 ± 21.86 | 1 | <0.001* | <0.001* |
| CRST C subscore | 14.55 ± 4.14 | 3.30 ± 3.34 | 2.05 ± 2.09 | 74.63 ± 26.13 | 41.35 ± 63.43 | 84.91 ± 14.62 | <0.001* | 0.308 | <0.001* |
| CRST C speech subscore* | 1.00 (0.00–2.00) | 0.00 (0.00-2.00) | 0.00 (0.00-1.00) | 100.00 (0.00-100.00) | 50.00 (0.00-100.00) | 58.33 (0.00-100.00) | 0.033* | 1 | 0.014* |
| QUEST score |  |  |  |  |  |  |  |  |  |
| Total score | 36.43 ± 16.48 | 33.40 ± 24.02 | 7.74 ± 8.15 | 10.45 ± 78.15 | 61.34 ± 47.27 | 75.64 ± 23.44 | 0.398 | 0.005* | <0.001* |
| Communication domain | 0.57 ± 1.45 | 1.35 ± 3.22 | 0.68 ± 1.83 | 0.00 ± 70.71 | 50.00 ± 57.74 | 75.00 ± 35.36 | 1 | 0.855 | 1 |
| Work/finances domain | 2.57 ± 3.37 | 3.00 ± 3.18 | 1.68 ± 4.16 | 0.38 ± 56.61 | 50.60 ± 89.70 | 72.92 ± 39.78 | 1 | 0.357 | 0.276 |
| Hobbies/leisure domain | 4.79 ± 4.30 | 2.55 ± 3.91 | 0.58 ± 2.29 | 69.17 ± 40.45 | 87.50 ± 35.36 | 100.0 ± 0.0 | 0.033* | 0.273 | 0.046* |
| Physical domain | 16.93 ± 11.70 | 16.80 ± 13.71 | 2.79 ± 4.47 | 93.25 ± 450.87 | 60.62 ± 68.96 | 80.65 ± 40.97 | 1 | 0.006* | 0.006* |
| Psychosocial domain | 11.57 ± 9.21 | 9.70 ± 19.14 | 2.00 ± 2.43 | 18.84 ± 104.06 | 62.97 ± 98.18 | 54.01 ± 112.57 | 0.099 | 0.012* | 0.015* |
| EQ5D |  |  |  |  |  |  |  |  |  |
| Self-reported health condition (%) | 67.14 ± 16.02 | 71.50 ± 12.68 | 84.71 ± 12.81 | 2.80 ± 14.86 | 22.46 ± 25.85 | 31.70 ± 31.31 | 1 | 0.002* | 0.003* |
| EQ5D (ISEV)^c^ | 20.70 ± 13.80 | 11.5 ± 12.70 | 8.90 ± 12.00 | 5.70 ± 14.00 | 1.10 ± 15.20 | 8.50 ± 17.20 | 0.501 | 1 | 0.213 |
| Berg Balance Scale^d*^ |  |  |  |  |  |  |  |  |  |
|  | 56 (56-56) | 56 (54-56) | 56 (55-56) | 0.00 (0.00-3.57) | 0.89 (0.00-2.76) | 0.00 (0.00-1.79) | 0.129 | 0.501 | 0.100 |

**Supplementary Table 2. Primary and Secondary Efficacy Outcomes. n=20.** *P*value <0.05

Continuous values are expressed in mean and standard deviation (SD).

^*^Non parametric distribution. Continuous values are expressed in median and interquartile range (IQR).

Timepoints: Baseline, FUS1 (6 months after the first thalamotomy) and FUS 2 (6 months after the second thalamotomy).

CRST: Clinical Rating Scale for Tremor. UL: Upper Limb. QUEST: Quality Of Life in Essential Tremor Questionnaire.

^a^Refers to the side treated in the first thalamotomy.

^b^Refers to the side treated in the second thalamotomy.

^c^EQ-5D 5L severity index. The severity index (ISEV) was calculated from the score of the individual scores of the 5 dimensions; It is a percentage on a scale 0-100%, where 0 represents the absence of severity and 100 the maximum possible severity.

^d^Berg Balance Scale (BBS) is a fourteen-item objective measure that assesses static balance and fall risk in adults. Higher scores reveal better balance (range 41-56 indicating independent walking). A score of less than 45 indicates that individuals may be at greater risk of falling.

| **Supplementary Table 3. Sonication related Adverse Events — no, of patients (%)** | | |
| --- | --- | --- |
|  | **1^st^ thalamotomy** | **2^nd^ thalamotomy** |
| Nausea | 0 (0) | 0 (0) |
| Vomiting | 0 (0) | 0 (0) |
| Dizziness | 3 (15) | 1 (5) |
| Headache | 0 (0) | 0 (0) |
| Head discomfort | 3 (15) | 1 (5) |
| Limb weakness | 1 (5) | 1 (5) |
| Paresthesia | 5 (25) | 7 (35) |
| Dysmetria | 1 (5) | 1 (5) |

**Supplementary Table 3. Sonication related Adverse Events**

^a^Patients who reported “unsteady gait” were those who reported having less equilibrium while walking.

**Supplementary Table 4. Preoperative MRI parameters**

|  | **T2-weighted** | **Reconstructed T2-weighted** |
| --- | --- | --- |
| **Image mode** | Three-dimensional | Three-dimensional |
| **TR (ms)** | 2500 | 2500 |
| **TE (ms)** | 240 | 240 |
| **Echo Train Length** | 112 | 112 |
| **No. of slices** | 626 | 150 |
| **Matrix size** | 320x320 | 512x512 |
| **Slice thickness (mm)** | 0.8 | 1.2 |
| **Slice gap (mm)** | 0.4 | 0.4 |
| **Bandwidth (Hz/px)** | 950 | 950 |
| **Field of view (mm)** | 250 | 250 |
| **Voxel Size (mm)** | 0.8x0.8x0.8 | 0.4x0.4x1.2 |

**Supplementary Table 4. Preoperative MRI parameters**

The table summarizes the acquisition parameters for preoperative MRI, comparing T2-weighted and reconstructed T2-weighted image modes. Both imaging modes use three-dimensional acquisition with identical TR (2500 ms), TE (240 ms), echo train length (112), and bandwidth (950 Hz/px). However, differences arise in matrix size (320x320 vs. 512x512), number of slices (626 vs. 150), slice thickness (0.8 mm vs. 1.2 mm), and voxel size (0.8x0.8x0.8 mm vs. 0.4x0.4x1.2 mm). Both methods maintain the same field of view (250 mm) and slice gap (0.4 mm). TR: Repetition Time. TE: Echo Time. ms: milliseconds. mm: millimetres. Hz: Herz. Px: Pixel.

| **Supplementary Table 5. Neuropsychological assessment** | | | |
| --- | --- | --- | --- |
|  | **FUS1** | **FUS2** | **P value** |
| **Memory** |  |  |  |
| T@M | 45.72 ± 16.22 | 45.06 ± 22.25 | 0.213 |
| RBANS - Immediate Memory | 95.24 ± 36.26 | 95.02 ± 47.31 | 0.318 |
| RBANS - Delayed Memory | 97.48 ± 35.83 | 96.18 ± 47.89 | 0.481 |
| **Language** |  |  |  |
| RBANS - Language | 98.55 ± 35.56 | 95.84 ± 47.48 | 0.446 |
| Semantic Fluency (animals) | 18.34 ± 8.90 | 17.09 ± 10.59 | 0.500 |
| **Visuospatial** |  |  |  |
| RBANS - visuospatial | 92.00 ± 34.89 | 79.88 ± 39.98 | 0.160 |
| Poppelreuter Test | 5.07 ± 1.80 | 5.00 ± 2.43 | 1 |
| Watch Test | 4.0 ± 1.40 | 4.09 ± 2.01 | 1 |
| **Attention and Executive Functions** | | | |
| RBANS - Attention | 79.44 ± 30.88 | 79.56 ± 41.16 | 0.178 |
| Stroop Naming | 77.99 ± 35.77 | 78.32 ± 41.33 | 0.330 |
| Stroop Color | 54.65 ± 25.05 | 54.54 ± 28.64 | 0.213 |
| Phonetic Fluency (F) | 9.18 ± 5.84 | 10.98 ± 7.76 | 0.065 |
| Phonetic Fluency (A) | 7.81 ± 4.54 | 10.25 ± 6.25 | 0.050 |
| Phonetic Fluency (S) | 9.36 ± 5.11 | 8.51 ± 6.15 | 0.429 |

**Supplementalry Table 5. Neuropsychological assessment**

Data are expressed in the patients' maximum scores on the evaluated tests. For all scales better performance is reflected by higher scores on each sub-item. Data are expressed in mean and standard deviations. T@M: Test de Alteración de Memoria (Spanish). RBANS: Repeatable Battery for the Assessment of Neuropsychological Status. *Pvalue < 0.05.
